# Health management practices in chronic traumatic brain injury rehabilitation: A scoping review protocol

**DOI:** 10.1371/journal.pone.0351635

**Published:** 2026-06-22

**Authors:** Davina Sharma, Shané J. Gill, Lizbeth Goodman, Amanda Rabinowitz, Louis N. Hunter, Reinhard Schaler, Kirby Wycoff

**Affiliations:** 1 Department of Health Sciences & Clinical Practice, Thomas Jefferson University, Philadelphia, Pennsylvania, United States of America; 2 University College Dublin, School of Mechanical and Materials Engineering, Newstead, Ireland; 3 Jefferson Moss Rehabilitation Research Institute, Thomas Jefferson University, Elkins Park, Pennsylvania, United States of America; 4 Jefferson College of Health Professions, Thomas Jefferson University, Philadelphia, Pennsylvania, United States of America; 5 An Saol Foundation, Dublin, Ireland; Federal University of Paraiba, BRAZIL

## Abstract

**Introduction:**

Traumatic brain injury is increasingly recognized as a chronic condition requiring long-term rehabilitation and coordinated care beyond the acute phase. Across Europe, substantial variation exists in healthcare system organization, funding structures, and service delivery models, which may influence access to rehabilitation and long-term functional outcomes for adults with chronic moderate-to-severe traumatic brain injury. The objective of this scoping review protocol is to systematically characterize systemic factors and management practices that shape rehabilitation access, continuity of care, and recovery outcomes across European healthcare systems. This review will map published evidence on health management practices influencing chronic traumatic brain injury rehabilitation in Europe and identify gaps for future research.

**Methods and Analysis:**

This review will follow the Joanna Briggs Institute methodology for scoping reviews. A comprehensive search of PubMed, Scopus, Ovid, and CINAHL will identify studies published between 2018 and 2025 involving adults aged 18 years or older who are at least 1-year post-injury. Studies examining health management practices (i.e., individual, organization, and systemic factors) within European healthcare context will be included. Two reviewers will independently screen articles using predefined inclusion criteria and documented using a Preferred Reporting Items for Systematic Reviews and Meta-Analyses extension for Scoping Reviews flow diagram. Data will be synthesized descriptively through tables, figures, and narrative summaries to map existing evidence and identify gaps in the literature.

## Background

### Conceptualization and clinical characteristics of traumatic brain injury

Traumatic brain injury (TBI) refers to damage to the brain caused by an external force. Once conceptualized as an injury with a finite recovery period, TBI is now recognized as a chronic condition with long-term consequences across multiple domains of health and function, some of which may deteriorate over time [1,2,3]. TBI severity is commonly classified using the Glasgow Coma Scale (GCS), a standardized measure of consciousness assessed within hours of injury that guides subsequent evaluation (e.g., neuroimaging) and acute management. In cases of more severe injury, early care appropriately prioritizes life-saving interventions and medical stabilization. However, this acute, unimodal indicator of injury severity maps poorly onto long-term recovery trajectories, which are highly heterogeneous even among individuals with similar early injury profiles [1,4]. Individuals with moderate-to-severe TBI (GCS score < 13) may experience delayed or progressive symptoms such as fatigue, cognitive dysfunction, emotional dysregulation, and late-emerging comorbidities, including depression or neurodegeneration [ 2,5–7]. The chronicity of TBI and emergent symptoms highlights the need for ongoing medical, psychological, and social support, underscoring rehabilitation as a central mechanism through which health systems address long-term recovery needs [1,8,2,9,10].

### Variability in European rehabilitation systems and access

Despite growing recognition of TBI as a chronic condition, requiring prolonged rehabilitation, care systems worldwide continue to prioritize acute care, leaving post-acute and long-term care markedly under-resourced [1,11]. For example, rehabilitation systems across Europe vary substantially by country in their organization, funding mechanisms, and availability of long-term services, shaping patient outcomes and quality of life [5,12]. Marklund et al. (2019) describe chronic TBI as involving neurological, behavioral, and systemic challenges, and recommended structured multidisciplinary pathways integrating neurology, rehabilitation, psychology, and social services to improve care continuity and long-term outcomes. Recently, Maas et al. (2022) confirmed earlier findings that individuals in Europe with moderate-to-severe TBI experience long-term functional impairments and late-emerging comorbidities, with little access to rehabilitation services as a continuous barrier to recovery. Jacob et al. (2020) demonstrated that systemic and geographic differences across Europe contribute to inequities in rehabilitation access, with some regions offering centralized public models and others relying on limited local services.

Variations in health system design and policy frameworks across Europe affect the quality and continuity of rehabilitation. Jourdan et al. (2019) compared TBI care pathways in Finland and France, revealing that national system organization affects both continuity of care and patient outcomes. Similarly, Gravesteijn et al. (2021) identified disparities in prehospital and emergency care infrastructure, including variation in early assessment practices, triage protocols, and access to specialized acute services, contributing to downstream inequities in chronic-phase rehabilitation. Lagares et al. [13] recently conducted a Delphi consensus study emphasizing the need for harmonized, evidence-based practices to standardize TBI care across Europe, especially for older adults with complex needs. The study focused on expert consensus; however, this scoping review will map published evidence on practice differences and identify gaps. Together, these findings demonstrate variability in care delivery and the need for comparative evidence synthesis on systemic and management factors shaping long-term outcomes.

### Role of social determinants in TBI recovery and rehabilitation access

Social determinants of health, including socioeconomic status, financial and geographic access to specialized rehabilitative care, and health system capacity for long-term services, influence recovery outcomes for individuals with chronic TBI across Europe [14–16,5]. Lavezzi et al. (2022) introduced a minimal rehabilitation assessment protocol emphasizing functional and psychosocial recovery to address disparities stemming from limited post-acute services, socioeconomic barriers, and regional variability in long-term rehabilitation services. Similarly, McElligott et al. (2011) described multidisciplinary European rehabilitation models that incorporate medical, psychological, and social supports within centralized public systems to reduce inequities related to income, geographic access, and service coordination. Furthermore, Hovset et al. (2024) note that patient-centered care and regular follow-up help mitigate social determinants by strengthening care continuity, enhancing engagement, and improving long-term recovery outcomes in individuals with chronic TBI. Despite efforts to improve coordination and access, variation in European TBI services produces socioeconomic and regional differences, underscoring the need to map impacts on rehabilitation continuity and long-term outcomes.

### Health management practices and evidence gaps in TBI rehabilitation

Although prior studies have examined the variability of chronic TBI care and social determinants of recovery that shape inequity in rehabilitation and health-related outcomes in European countries, no study has synthesized these determinants and explored rehabilitative and functional outcomes across European health systems. Existing literature focuses on isolated determinants of access or satisfaction, with researchers not accounting for how national or regional health system structures collectively influence long-term outcomes among persons diagnosed with chronic-phase TBI. Few studies synthesize evidence on how systemic and management practices, such as care coordination models, policy frameworks, or rehabilitation intensity, affect recovery trajectories in chronic-phase TBI across Europe. Mapping this evidence is critical for identifying key systemic and organizational barriers and outlining the changes needed to improve continuity and quality of life for individuals with TBI. Therefore, this review will contribute to the literature by describing the extent and nature of available evidence on health management factors affecting rehabilitation access, delivery, and outcomes during the chronic-phase of TBI in Europe.

Despite recognition of multilevel factors shaping long-term outcomes following a TBI in European countries, a key factor that contributes to inequitable rehabilitation and functional outcomes are health management practices [17]. Studies across Europe have demonstrated substantial variation in health management practices, including differences in rehabilitation pathways, multidisciplinary coordination, discharge planning, and access to specialized rehabilitation services following TBI [ 9,12], with practitioners noting “different organizations” and “common issues” in care pathways across countries [9 (pp205)]. For example, practitioners in Finland and France identified differences in organizational structures and continuity of care pathways that contributed to inconsistent access to rehabilitation services and long-term support following TBI. Additionally, broader European studies found that disparities in rehabilitation access were associated with variations in healthcare infrastructure, resource allocation, and system-level coordination across countries [5,12], including unequal access to services within the first year post-injury [5]. These differing health management practices have important implications for health equity, as disjointed or poorly coordinated rehabilitation systems and limited interdisciplinary collaboration may contribute to poorer functional outcomes, reduced patient satisfaction, and inequitable access to long-term rehabilitation supports among vulnerable populations [ 8,14], with caregivers reporting “barriers” to accessing needed services [2 (pp130)] and patients’ satisfaction reflecting variability in service quality [8]. To investigate the effects of health management practices on rehabilitation outcomes among persons with TBI, a universal definition of this construct is warranted, offering a standard for comparison of practices and differential outcomes across Europe. As such, we define health management practices using the conceptual framework provided by the European Health Management Association (EHMA). According to the EHMA, health management practices include the “practice of providing guidance and leadership to promote and support health at the individual, organizational and systemic levels [18(pp790)].” This definition requires use of a total health approach in which we consider behavioral, social, and environmental indicators across all factors of health management practices [18] that further shape rehabilitation and functional outcomes. Pre-injury factors such as medical conditions or ailments and mental health diagnoses are prominent in literature examining the influence of pre-existing conditions that influence TBI care and outcomes over the life span [19,20]. Pre-injury problem-focused and emotional coping ability predict adjustment post-injury in persons with a TBI [20]. The social indicator consists of social support or psychosocial functioning [21,22–24]. Across these studies, researchers describe the variability in social support networks in persons with TBI, calling for the increase in quality and amount of support to improve psychological functioning, adjustment, and activity in this population. In addition, Kersey et al. (2024) acknowledged that persons with TBI may value different types of support compared to the general population, warranting adaptations to existing models to align with patient values. Therefore, using consistent themes in the literature on individual-level influences on TBI rehabilitation and outcomes, we define individual indicators of health management practices as demographic characteristics (e.g., age, sex, and gender), pre-injury factors, social networks, and culture. This definition was adopted due to the indicator not being defined explicitly within the conceptual framework. Similarly, systemic factors were not defined. Therefore, we define systemic factors as foundational and structural components that influence how services are structured, financed, and delivered to all citizens, which addressing macro-level concepts that shape equity and quality of care [25] .

### Study objectives and rationale

The objectives of this scoping review study are to (1) identify individual, organizational, and systemic factors within health management practices that influence rehabilitation and functional outcomes during the chronic phase of TBI and (2) to describe how variability in health management practices across European countries shapes health inequity and quality of care. This review will describe how health management practices shape access, service continuity, and long-term recovery, identifying gaps for future research. A scoping review is appropriate to map the extent, heterogeneity, and emerging nature of the evidence on chronic TBI rehabilitation and system-level influences across Europe [26]. This method supports a comprehensive synthesis of quantitative and qualitative data to capture how structural, organizational, and policy-related factors influence long-term TBI outcomes.

A preliminary search of Medical Literature Analysis and Retrieval System Online (MEDLINE), the Cochrane Database of Systematic Reviews, and Joanna Briggs Institute (JBI) evidence identified no existing or ongoing scoping or systematic reviews on this topic.

## Methods

This scoping review protocol outlines the planned approach to mapping existing evidence on health management practices influencing rehabilitation and functional outcomes during the chronic phase of traumatic brain injury (TBI) across European healthcare systems. The scoping review will be conducted in accordance with the Joanna Briggs Institute (JBI) Scoping Review Framework. Reporting will follow the Preferred Reporting Items for Systematic Reviews and Meta-Analyses extension for Scoping Reviews (PRISMA-ScR) to ensure transparency and completeness. The protocol was registered with the Open Science Framework (OSF) on Feb 10, 2026, [DOI: https://doi.org/10.17605/OSF.IO/RFEKY] ensuring transparency and reproducibility of the review process. Ethics approval is not required because the review uses published literature.

### Stage 1: Clarifying and linking the purpose and research questions

Preliminary exploration of the literature revealed substantial variation in how chronic-phase rehabilitation, continuity of care, and long-term functional outcomes following moderate-to-severe traumatic brain injury (TBI) are conceptualized and reported across European healthcare systems. Existing studies frequently examine system-level factors (e.g., funding, policies, referral pathways) separately from management practices (e.g., care coordination, rehabilitation models service delivery, intensity), limiting a comprehensive understanding of how these elements collectively influence long-term rehabilitation outcomes.

To address this gap and ensure methodological clarity, the review question was developed in alignment with the Population, Concept, and Context (PCC) framework. The components were defined as follows:

- Population: Adults 18 years or older with moderate-to-severe TBI in the chronic phase (≥1-year post-injury).
- Concept: Health management practices (i.e., individual, organizational, systemic) influencing rehabilitation delivery and functional outcomes as defined in the introduction.
- Context: European healthcare systems.

Guided by this framework, the review is structured around the following primary question:

1 What health management practices influence rehabilitation and functional outcomes during the chronic phase of TBI in Europe?

The secondary question is:

2 How do these factors vary across European countries, and how do they contribute to differences in long-term recovery outcomes?

The objectives of this study are to (1) identify individual, organizational, and systemic factors within health management practices that influence rehabilitation and functional outcomes during the chronic phase of TBI and (2) to describe how variability in health management practices across European countries shapes health inequity and quality of care. These research questions provide a clear foundation for identifying relevant evidence and ensure the review remains focused on the interplay between health management practices and patient outcomes in chronic-phase rehabilitation in Europe.

### Stage 2: Identifying relevant studies

A comprehensive search strategy will be developed to identify studies that directly address the research questions. An initial search of PubMed, CINAHL, Scopus and OVID MEDLINE, will be conducted to identify relevant keywords and index terms. Terms will be organized according to the Population, Concept, and Context (PCC) framework to ensure alignment with the review objectives.

The finalized search strategy will be adapted for PubMed, CINAHL, Scopus, and Ovid MEDLINE. The search will include studies published between January 2018 to December 2025 to capture contemporary rehabilitation practices. Studies must involve adults aged 18 years or older who are at least one-year post-injury and conducted within European healthcare settings. Due to resource constraints, only English-language publications will be included; this may limit representation of non-English European literature. Reference lists of included studies will also be screened.

By systematically identifying studies that address healthcare management practices and rehabilitation models, this search strategy provides extensive coverage of the current evidence base on chronic-phase traumatic brain injury rehabilitation across Europe.

### Stage 3: Study screening and selection

Following the search, all identified citations will be collated and imported into Covidence systematic review software, where duplicates will be removed. Consistent with established scoping review methodology and PRISMA-ScR reporting standards, a structured and calibrated screening process will be employed to ensure methodological rigor and reduce reviewer bias. Two reviewers will independently screen titles and abstracts against the predefined inclusion and exclusion criteria. Prior to initiating full title-and-abstract screening, reviewers will pilot-test a random sample of 25 citations to ensure consistent interpretation of the eligibility criteria. Discrepancies identified during pilot screening will be discussed and resolved by a third reviewer. Full-text screening will then be conducted independently by two reviewers. Any disagreements will be resolved with a third reviewer. Reasons for exclusion at the full-text stage will be documented to ensure transparency. The study selection process will be summarized using a PRISMA-ScR flow diagram, detailing the number of records identified, screened, excluded, and included.

### Stage 4: Charting the data

Data will be extracted independently by two reviewers using a structured charting tool developed by the research team in accordance with established scoping review methodology. The extraction tool is designed to align with the Population, Concept, and Context framework and the review’s primary and secondary research questions. A draft charting form is provided in Appendix II. Extracted data will include citation details, year of publication, country of study, study aims, design and methodology, participant characteristics, TBI severity and chronicity, descriptions of healthcare system structures, health management practices (individual, organizational, and systemic indicators), rehabilitation models, reported functional or quality-of-life outcomes, key findings relevant to the review questions, and reported study limitations. The extraction tool will be piloted on a subset of included studies to ensure consistent interpretation and to confirm that relevant variables are sufficiently captured. The data extraction tool will be refined as necessary, and any modifications will be documented in the final review. Discrepancies between reviewers will be resolved by a third reviewer when required. Consistent with scoping review methodology, critical appraisal of included studies will not be conducted.

### Stage 5: Collating, summarizing, and reporting the results

Once data have been extracted using the charting tool developed in Stage 4, the findings will be synthesized descriptively to provide a comprehensive overview of chronic-phase TBI rehabilitation across European healthcare systems. Data will be presented in tables and figures to map individual, organizational, and systemic indicators that influence access, continuity, and delivery of rehabilitation services, demonstrating consistency across Europe and variability in these indicators by country. Rehabilitation models, management approaches, and long-term support structures will also be summarized, along with functional and quality-of-life outcomes reported in the literature. This visual presentation will allow for the identification of patterns, similarities, differences, and gaps across European contexts. A narrative summary will accompany all tabulated and charted data, highlighting how the evidence addresses the review’s primary and secondary questions. This summary will provide a coherent interpretation of the findings, explaining how health management factors interact to shape rehabilitation outcomes, and will identify areas where evidence is limited or inconsistent, indicating priorities for future research. Consistent with scoping review methodology, this review will not evaluate intervention effectiveness or conduct a critical appraisal of included studies.

### Stage 6: Implications and dissemination

As this scoping review uses publicly available literature, ethics approval is not required. Findings will contribute to the evidence base by mapping health management factors influencing chronic-phase TBI rehabilitation in European healthcare systems, identifying existing gaps, and highlighting areas for further research. Results will be disseminated through peer-reviewed publications and presentations at scientific conferences to inform researchers, clinicians, and stakeholders about the current state of evidence, trends, and priorities for future studies.

## Supporting information

S1 File Search strategy. (DOCX)

S1 Table Data extraction instrument. (DOCX)

S2 File PRISMA-P-checklist. (DOCX)
