# Supplementary material for: Health management practices in chronic traumatic brain injury rehabilitation: A scoping review protocol
Source: PLoS One. 2026 Jun 22;21(6):e0351635. doi: 10.1371/journal.pone.0351635 (PMC13286134; doi:10.1371/journal.pone.0351635)
Supplement: S1 File — (DOCX) [file pone.0351635.s001.docx]

# **Supporting Information**

### **S1 File. Search strategy.**

The following search strategies were developed in collaboration with the assigned librarian and the OPWP&C reviewer. A full reproducible search strategy is presented for PubMed, with additional strategies for Scopus, Ovid MEDLINE, and CINAHL. All searches applied limits for publication year (2018–2025) and English language.

**Database: PubMed**

**Search String:** (("Brain Injuries, Traumatic"[Mesh] OR "Traumatic Brain Injury" OR "Traumatic Brain Injuries" OR TBI) AND ("Europe" [Mesh] OR "Ireland" [Mesh] OR Ireland OR "United Kingdom"[Mesh] OR United Kingdom OR "England"[Mesh] OR England OR "Northern Ireland"[Mesh] OR Northern Ireland OR "Scotland"[Mesh] OR Scotland OR "Wales"[Mesh] OR Wales OR "France"[Mesh] OR France OR "Germany" OR Germany OR "Gibraltar"[Mesh] OR Gibraltar OR "Greece"[Mesh] OR Greece OR "Italy"[Mesh] OR Italy OR "Liechtenstein"[Mesh] OR Liechtenstein OR "Luxembourg"[Mesh])) AND (Chronic Phase OR "Long-Term Care"[Mesh] OR Long-Term Care OR Post-Acute Rehabilitation OR "Diagnosis"[Mesh] OR Diagnosis OR "Delivery of Health Care"[Mesh] OR Delivery of Health Care OR "Chronic Care Model"[Mesh] OR Chronic Care Model) AND (2018:2025[pdat])

**Filters to apply in PubMed:** English and publication dates 2018-2025

**Records retrieved: 2823**

**Database: Scopus**

**Search String:** ( TITLE-ABS-KEY ( ( BRAIN INJURIES TRAUMATIC ) OR ( TRAUMATIC BRAIN INJURY ) OR ( TRAUMATIC BRAIN INJURIES ) OR ( TBI ) ) AND TITLE-ABS-KEY ( ( EUROPE ) OR ( IRELAND ) OR ( UNITED KINGDOM ) OR ( ENGLAND ) OR ( NORTHERN IRELAND ) OR ( SCOTLAND ) OR ( WALES ) OR ( FRANCE ) OR ( GERMANY ) OR ( GIBRALTAR ) OR ( GREECE ) OR ( ITALY ) OR ( LIECHTENSTEIN ) OR ( LUXEMBOURG ) ) AND TITLE-ABS-KEY ( ( CHRONIC PHASE ) OR ( LONG-TERM CARE ) OR ( POST-ACUTE REHABILITATION ) OR ( DIAGNOSIS ) OR ( DELIVERY OF HEALTH CARE ) OR ( CHRONIC CARE MODEL ) ) ) AND PUBYEAR > 2017

**Filters to apply in Scopus:** English and publication dates 2018-2025

**Records retrieved: 264**

**Database: OVID MEDLINE**

**Search String: 1 AND 2 AND 3:** (Brain Injuries, Traumatic/ or Brain Injuries Traumatic or Traumatic Brain Injury or Traumatic Brain Injuries or TBI) AND (Europe/ or Ireland/ or Northern Ireland/ or United Kingdom/ or England/ or Scotland/ or Wales/ or France/ or Germany/ or Gibraltar/ or Greece/ or Italy/ or Europe or Ireland or United Kingdom or England or Northern Ireland or Scotland or Wales or France or Germany or Gibraltar or Greece or Italy or Liechtenstein or Luxembourg) AND (Long-Term Care/ or Diagnosis/ or "Delivery of Health Care"/ or Chronic Care Model/ or Chronic Phase or Long-Term Care or Post-Acute Rehabilitation or Diagnosis or Delivery of Health Care or Chronic Care Model)

**Filters to apply in OVID MEDLINE:** Limiter: 1 to yr “2018-current” and English

**Records retrieved: 192**

**Database: CINHAL**

**Search String:** (( Brain Injuries Traumatic ) OR ( Traumatic Brain Injury ) OR ( Traumatic Brain Injuries ) OR ( TBI )) AND ((Europe) OR (Ireland) OR (United Kingdom) OR (England) OR (Northern Ireland) OR (Scotland) OR (Wales) OR (France) OR (Germany) OR (Gibraltar) OR (Greece) OR (Italy) OR (Liechtenstein) OR (Luxembourg)) AND ((Chronic Phase) OR (Long-Term Care) OR (Post-Acute Rehabilitation) OR (Diagnosis) OR (Delivery of Health Care) OR (Chronic Care Model))

**Filters to apply in CINHAL:** English and publication dates 2018-2025

**Records retrieved: 91**
