# Supplementary material for: Health management practices in chronic traumatic brain injury rehabilitation: A scoping review protocol
Source: PLoS One. 2026 Jun 22;21(6):e0351635. doi: 10.1371/journal.pone.0351635 (PMC13286134; doi:10.1371/journal.pone.0351635)
Supplement: S1 Table — (DOCX) [file pone.0351635.s002.docx]

### **Supporting Information**

### **S1 Table. Data extraction instrument.**

This charting tool maps to JBI’s recommended extraction fields and includes the metadata PRISMA-ScR expects (citation, design, country, participants, context, results).

| Field | Description / Data to Extract |
| --- | --- |
| 1. Citation | Full reference (author(s), year, title, journal, volume, issue, pages). |
| 2. Contact Information | Corresponding author’s email or institutional contact (if available). |
| 3. Country / Territory | Country or region where the study took place (e.g., Northern Europe, Ireland). |
| 4. Context / Setting | Healthcare setting (e.g., inpatient rehab unit, outpatient clinic, community-based rehab, NGO). |
| 5. Study Aim / Objective | Aim(s) or research question(s) stated by the authors. |
| 6. Study Design / Methodology | Study type (qualitative, quantitative, mixed methods) and design specifics (e.g., RCT, cohort, survey, interviews, observational study). |
| 7. Participants / Population | Patient and provider characteristics: TBI severity, chronicity, age, sex, number of participants, provider type(s) (e.g., neurologist, nurse, therapist). |
| 8. Rehabilitation / Management Practices | Type(s) of interventions or rehabilitation programs used (e.g., cognitive rehab, physical therapy, community reintegration, tele-rehab). |
| 9. Intervention Implementation | How, by whom, and how often interventions were delivered (frequency, duration, setting). |
| 10. Functional / Quality-of-Life Outcomes | Measured outcomes (e.g., functional independence, cognitive recovery, participation, quality of life) and how assessed. |
| 12. Systemic Factors | Structural, organizational, or resource-related influences on care delivery (e.g., cost, staffing, infrastructure, policy, access barriers). |
| 13. Key Findings | Main results relevant to chronic TBI rehabilitation and the review questions. |
| 14. Organizational Context Factors | Organizational conditions or resources that shape rehabilitation access, coordination, and long-term support (e.g., community service availability, coordination infrastructure, caregiver support systems). |
| 15. Authors’ Conclusions / Recommendations | Conclusions and recommendations reported by study authors (clinical, policy, or research implications). |
| 16. Limitations | Limitations reported by study authors. |
| 17. Relevance to Review Questions | How the study informs the scoping review objectives and questions. |
| 18. Reviewer Notes | Observations, data quality notes, or queries raised during extraction; any modifications made during extraction. |
