# Supplementary material for: Health management practices in chronic traumatic brain injury rehabilitation: A scoping review protocol
Source: PLoS One. 2026 Jun 22;21(6):e0351635. doi: 10.1371/journal.pone.0351635 (PMC13286134; doi:10.1371/journal.pone.0351635)
Supplement: Attachment — Submitted filename: Response to Reviewers.docx [file pone.0351635.s005.docx]

**Manuscript ID:** PONE-D-26-08745
**Manuscript Title:** *Health Management Practices in Chronic Traumatic Brain Injury Rehabilitation: A Scoping Review Protocol*

Friday, May 15^th^, 2026

Dear Reviewers,

On behalf of our research team, we would like to extend our sincerest gratitude for a thorough review and substantive feedback that we received on our manuscript *“Systemic factors and management practices in chronic traumatic brain injury rehabilitation: A scoping review protocol”.* We appreciate the positive evaluation of the manuscript’s structure, methodological rigor, and relevance of our scoping review protocol on traumatic brain injury (TBI) rehabilitation and management. We have carefully addressed the reviewer’s comment and revised the manuscript accordingly. Below, we provided a response to your comment and outline the corresponding revisions made to the manuscript (included in the second page of this letter in blue). Please accept this revision and an outline of feedback provided by all reviewers. We appreciate the opportunity to improve the clarity and conceptual consistency of this work and to strengthen its contribution to the field.

The title has been updated to “Health Management Practices in Chronic Traumatic Brain Injury Rehabilitation: A Scoping Review Protocol” to align with the revised manuscript and reflect the clarified emphasis on individual, organizational, and system-level factors.

Sincerely,

Research Team

**REVIEWER 1 MINOR REVISION-**

- **Comment from Reviewer #1: Syed Sajid Husain Kazmi:** The manuscript is structured and well written. It is methodologically sound and addresses important areas related to traumatic brain injury, its management and rehabilitation aspects. The manuscript may further be enhanced by explaining the 'systemic factors' and 'management practices' highlighted multiple times in the manuscript but not explained appropriately.
  - **Response:** In response, we have revised the manuscript to improve conceptual clarity by introducing and consistently applying a clearly defined framework centered on *health management practices* (in the background section (lines 158-205), and methods section (lines 237-249)). This construct is defined as a multi-level factor encompassing individual, organizational, and system-level indicators that influence rehabilitation delivery and functional outcomes in chronic traumatic brain injury.
    - To ensure consistency and reduce ambiguity, the term *“systemic factors”* has been removed throughout the manuscript and replaced with the more precise construct of *health management practices*. This revision aligns terminology across the introduction, eligibility criteria, data extraction tool, and analysis plan.
    - Where prior literature did not provide standardized definitions, we synthesized and operationalized existing evidence to establish uniform definitions for the components of health management practices. Specifically:
      - **Individual-level indicators** include demographic characteristics, pre-injury factors, social support, and culture.
      - **Organizational-level indicators** include rehabilitation service delivery models, coordination structures, and resource availability.
      - **System-level indicators** include healthcare policy frameworks, funding structures, access pathways, and system-level organization of rehabilitation services.
